# LDL cholesterol estimation in patients with the metabolic syndrome

**DOI:** 10.1186/1476-511X-5-8

**Published:** 2006-04-06

**Authors:** Irene Gazi, Vasilis Tsimihodimos, Theodosios D Filippatos, Vasilios G Saougos, Eleni T Bairaktari, Alexandros D Tselepis, Moses Elisaf

**Affiliations:** 1 Department of Internal Medicine, Medical School, University of Ioannina, Ioannina, Greece; 2 Laboratory of Biochemistry, Medical School, University of Ioannina, Ioannina, Greece; 3 Laboratory of Biochemistry, Department of Chemistry, University of Ioannina, Ioannina, Greece

## Abstract

**Background:**

The Friedewald formula (LDL-F) for the estimation of low-density lipoprotein (LDL) cholesterol concentrations is the most often used formula in clinical trials and clinical practice. However, much concern has been raised as to whether this formula is applicable in all patient populations such as the presence of chylomicronaemia and/or hypertriglyceridaemia. The aim of the present study was to evaluate various LDL cholesterol calculation formulas as well as LDL cholesterol levels provided by the Lipoprint LDL System (LDL-L) in patients with the metabolic syndrome (MetSyn).

**Results:**

LDL-F showed significant differences from other formulas in the total cohort, as well as in MetSyn individuals. This was not the case in nonMetSyn subjects, where LDL-F did not differ with other formulas, with the exception of one formula (LDL by Planella, LDL-P). The bias between LDL-F and other LDL estimation formulas were significantly higher in MetSyn subjects compared to nonMetSyn individuals, except for LDL-L which produced similar bias with LDL-F in both study groups.

**Conclusion:**

LDL-F seems to exhibit some limitations as far as the calculation of LDL-C levels in patients with the MetSyn is concerned. LDL-L might be more accurate in MetSyn subjects, but so far its use is limited for the estimation of small, dense LDL (sdLDL) cholesterol levels and mean LDL particle size for research purposes only.

## Background

The association between total cholesterol (TC) and the risk of atherosclerotic disease is well established by many studies, such as the Framingham Heart Study [1]. Low-density lipoprotein (LDL) particles are the main carriers of the circulating cholesterol and they play a key role in cholesterol transfer and metabolism [2]. The Third Report of the National Cholesterol Education Program (Adult Treatment Panel III) (NCEP ATP III) guidelines focus on LDL-cholesterol (LDL-C) levels as the primary target of cholesterol-lowering therapy [3]. Friedewald formula is the most often used formula for the calculation of LDL-C levels in clinical trials [4]. However, much concern has been raised as to whether this formula is applicable in all patient groups [5].

The principle of the Friedewald formula (LDL-F) is based on two assumptions: Firstly, that since very low-density lipoprotein (VLDL) carries most of the circulating triglycerides (TG), VLDL-cholesterol (VLDL-C) can be estimated reasonably well as TG concentrations divided by constant (i.e. 5 for mg/dL and 2.2 for mmol/L) [2,5]. Secondly, that TC is distributed among the three major lipoprotein subclasses [namely high-density lipoprotein (HDL), LDL and VLDL] [2]. There are though some limitations as to the calculation of LDL-C according to the LDL-F formula, the two most common being that the patient needs to be fasting for at least 12 hours and that TG levels have to be <400 mg/dL [6].

Metabolic syndrome (MetSyn) comprises a clustering of atherosclerotic factors, including abdominal obesity, dyslipidemia, disturbed carbohydrate metabolism, and hypertension [7]. The NCEP ATP III criteria for the diagnosis of the MetSyn are as follows: waist circumference >102 cm in men and >88 cm in women, TG ≥ 150 mg/dL, high-density lipoprotein cholesterol (HDL-C) < 40 mg/dL in men and < 50 mg/dL in women, fasting glucose ≥ 110 mg/dL and blood pressure ≥ 130/85 mm Hg (or use of antihypertensive agents) (three of the previously mentioned criteria are required for the diagnosis of the Met Syn) [3]. Data from the ATTICA and the METS-GREECE studies showed that the overall prevalence of the MetSyn in the Greek population is 19.8% and 23.6%, respectively [8-10]. The most common lipid abnormalities associated with the MetSyn are elevated TG levels, low HDL-C levels and the existence of small dense LDL (sdLDL) particles [11]. SdLDL particles are thought to be more atherogenic than large, buoyant particles [12,13]. Large clinical studies have shown a consistent association between sdLDL particles and incident atherosclerotic disease [14,15].

The purpose of the present study was to evaluate the accuracy of various formulas for the calculation of LDL-C levels in patients with the MetSyn.

## Results

Of the total cohort, 105 subjects fulfilled the NCEP ATP III MetSyn criteria, whereas 74 individuals did not. As shown in Table 1, MetSyn subjects exhibited higher levels of TC, TRG, and apoB and lower HDL-C and apoA-I levels compared to individuals not fulfilling the criteria for the diagnosis of MetSyn.

**Table 1:** Basic lipid profile of the study participants.

Parameters	Total (n = 179)	MetSyn (n = 105)	nonMetSyn (n = 74)
TC (mg/dL)	229 ± 43	238 ± 45	218 ± 37*
TG (mg/dL)	162 ± 82	196 ± 77	113 ± 62*
HDL-C (mg/dL)	52 ± 11	49 ± 10	57 ± 12*
apoA-I (mg/dL)	138 ± 25	131 ± 25	148 ± 22*
apoB (mg/dL)	104 ± 26	109 ± 27	96 ± 24*

TC: total cholesterol, TG: triglycerides, HDL-C: high-density lipoprotein cholesterol, apo: apolipoprotein

Data represent mean ± SD.

*p ≤ 0.001 compared to MetSyn subjects.

Table 2 shows the estimation of LDL-C according to various formulas in the total cohort, as well as in MetSyn and nonMetSyn subjects. LDL-C levels were significantly but borderline different between the two study groups when LDL-C was calculated according to the formulas proposed by Friedewald (LDL-F) and by Hattori (LDL-H) (p = 0.04 for both comparisons), whereas it was significantly different when it was calculated according to the formula used in AMORIS study (LDL-A) (p = 0.001). It should be noted that no differences were detected between MetSyn and nonMetSyn subjects when LDL-C was calculated according to the formulas proposed by Planella and Wagner (LDL-P and LDL-W, respectively) and when LDL-C levels were provided by the Lipoprint System (LDL-L). When LDL-C estimations were adjusted for TG levels (to avoid the confounding effect of TG on LDL determination), no differences were observed between MetSyn and nonMetSyn groups for any LDL-C estimation formula (data not shown).

**Table 2:** LDL-cholesterol concentrations calculated according to various formulas in the total cohort, as well as in MetSyn and nonMetSyn subjects

LDL-C estimated	Total (n = 179)	MetSyn (n = 105)	nonMetSyn (n = 74)	p (between MetSyn and nonMetSyn subjects)
LDL-F	145 ± 35	149 ± 38	138 ± 28	0.04
LDL-P	129 ± 29*	132 ± 32*	126 ± 26**	NS
LDL-H	136 ± 33**	140 ± 36	130 ± 27	0.04
LDL-A	145 ± 41	154 ± 44	132 ± 33	0.001
LDL-W	138 ± 30	140 ± 33	136 ± 26	NS
LDL-L	133 ± 31*	135 ± 30**	130 ± 32	NS

Data are expressed as mean ± SD.

LDL: low-density lipoprotein, MetSyn: metabolic syndrome, LDL-F: LDL-cholesterol by Friedewald, LDL-P: LDL-cholesterol by Planella, LDL-H: LDL-cholesterol by Hattori, LDL-A: LDL-cholesterol by AMORIS, LDL-W: LDL-cholesterol by Wagner, LDL-L: LDL-cholesterol by the Lipoprint LDL system. One-way analysis of variance (ANOVA) followed by Least Significance Difference (LSD) for paired comparisons *p < 0.001, **p ≤ 0.01 compared to LDL-F.

Additionally, in the total cohort significant differences were found between LDL-C estimations (overall p < 0.001). Specifically, as shown in Table 2, LDL-F was significantly higher than LDL-P, LDL-H and LDL-L (p < 0.001, = 0.01 and <0.001, respectively). In the MetSyn group, significant differences were also observed between various LDL-C estimations (overall p < 0.001), due to the differences observed between LDL-F and LDL-P (p < 0.001) and LDL-F and LDL-L (p < 0.01) (Tables 2 and 3). Interestingly, in the nonMetSyn group the only difference observed was between LDL-F and LDL-P (p < 0.01) (Tables 2 and 3).

**Table 3:** Mean differences between LDL-F and other LDL-C estimations in the total cohort, as well as in MetSyn and nonMetSyn individuals.

	Mean difference (mg/dL) (bias %)		
	
	Total (n = 179)	MetSyn (n = 105)	nonMetSyn (n = 74)
(LDL-F) – (LDL-P)	14 (-16–64)	16 (-16–64)	13 (-6–27)*
	10 (-13–33)	11 (-13–33)	9 (-5–18)**
(LDL-F) – (LDL-H)	9 (3–16)	10 (3–16)	8 (6–13)*
	6 (3–7)	6 (6–7)	6 (6–7)*
(LDL-F) – (LDL-A)	0.5 (-48–28)	-4 (149-28)	8 (-27-24)*
	0.5 (-31–35)	-3 (-31–35)	6 (-26-20)*
(LDL-F) – (LDL-W)	4 (-30–62)	7 (-30–62)	3 (-17–18)*
	3 (-25–33)	4 (-25–33)	3 (-16-11)**
(LDL-F) – (LDL-L)	16 (-48–70)	14 (-47–70)	16 (-48–51)
	10 (-48-37)	9 (-47-37)	12 (-48-33)

Data are expressed as median (range).

LDL: low-density lipoprotein, MetSyn: metabolic syndrome, LDL-F: LDL-cholesterol by Friedewald, LDL-P: LDL-cholesterol by Planella, LDL-H: LDL-cholesterol by Hattori, LDL-A: LDL-cholesterol by AMORIS, LDL-W: LDL-cholesterol by Wagner, LDL-L: LDL-cholesterol by Lipoprint LDL system. Mann-Whitney U test *p < 0.01 and **p < 0.05 compared to MetSyn individuals.

Table 3 shows the biases (absolute values and percentages) observed between LDL-F and all other LDL estimations. The biases between LDL-F and all other LDL-C estimations were different between MetSyn and nonMetSyn subjects except for LDL-L which did not differ between the two study groups (Table 3).

We subsequently evaluated the correlation coefficients between the various LDL formulas in the total cohort, as well as within each study group (data not shown). All correlations were statistically significant in all study groups (p < 0.001 for all correlations), but there were some discrepancies between formulas. LDL-L in the total cohort showed the least correlation with all other formulas (Pearson's r ranged between 0.707 and 0.787, whereas all other r were above the value of 0.83) (data not shown).

Finally we estimated the predictive power of the ratio LDL-H/LDL-apoB to identify individuals with decreased mean LDL particle size. We used as cut-off point the value of 268Å of mean LDL particle size to identify subjects with decreased LDL size, according to the manufacturer's instructions. Hattori et al proposed that a value of 1.2 or less of the LDL-H/LDL-apoB ratio postulates the preponderance of sdLDL particles. Interestingly, the sensitivity and specificity of this ratio to identify individuals with decreased mean LDL particle size were 2.6% and 96.9%, respectively and the positive and negative predictive values were 60% and 36.2%, respectively (data not shown).

## Discussion

The association between elevated LDL-C levels and increased risk of atherosclerotic disease has been clearly demonstrated. Currently, most clinical laboratories and clinical studies use the Friedewald formula for the estimation of LDL-C levels, since the reference method, i.e. β-quantification by ultracentrifugation, is not suitable for routine use [4,6]. However, the accuracy of Friedewald formula has been questioned in many patient groups, such as patients with hypertriglyceridaemia [5], with type III hyperlipidaemia [2], as well as in patients with secondary hyperlipidaemias, such those observed in patients with diabetes mellitus, renal disease, hepatic failure, as well as those on hormone replacement therapy [6,16,17]. These conditions are characterized not only by increased TG levels but also by lipoprotein alterations, indicating that the acceptable reliability of the LDL-F formula at TG concentrations of 200–400 mg/dL needs special concern in selected patient populations [2].

Dyslipidaemia is a hallmark of the MetSyn. The underlying mechanism of the dyslipidaemia of the MetSyn is the altered metabolism of TG-rich lipoproteins, such as VLDL and IDL remnants [7,11]. Except for being a major component of MetSyn, elevated TG levels are also very common in the general population [18]. This finding strengthens the need of a formula for the estimation of LDL-C levels that is applicable in individuals with raised TG levels.

The present study is -to our knowledge- the first study to evaluate various LDL-C calculation formulas in subjects with the MetSyn. Moreover, a new LDL-C estimation is presented for the first time, i.e. LDL-L.

The estimation of LDL-C levels according to LDL-L method showed in the total population a mean difference with LDL-F [16(-48–70) mg/dL] approaching the previously reported difference between LDL-F and LDL measured by ultracentrifugation (LDL-UC) ranging between 6.8 ± 13.3 mg/dL and 13.5 ± 13.9 mg/dL [6,16,17]. Moreover, the difference observed between LDL-F and LDL-P, as well as between LDL-F and LDL-H in the total cohort [14(-15–64) mg/dL and 9(3–16) mg/dL, respectively] approached the difference observed between LDL-F and LDL-L. This finding indicates that these methods for the estimation of LDL-C levels may approach the accuracy of the gold standard method for the evaluation of LDL-C levels, i.e β-quantification by ultracentrifugation. In MetSyn subjects the bias between LDL-F and LDL-L was 9(-47-37)% and between LDL-F and LDL-P it was 11(-13–33)%, whereas the corresponding differences in the nonMetSyn group were 12(-48-34)% and 9(-5–19)% respectively. Moreover, the bias between LDL-F and LDL-H was 10(3–16) mg/dL in the MetSyn group and 8(6–13) mg/dL in the nonMetSyn group. The bias between LDL-F and LDL-L did not differ between MetSyn and nonMetSyn subjects while the biases between LDL-F and LDL-P and LDL-F and LDL-H were higher in the MetSyn group compared to the nonMetSyn group.

ApoB levels are representative of the number of apoB containing particles, even if alterations of their lipid content is present, as occurs in subjects with elevated sdLDL particles [17]. This pattern is often seen in patients with diabetes mellitus, with the MetSyn as well as in patients with elevated TG levels, even if LDL-C concentrations are relatively low [7,11]. Moreover, it has been proposed that individuals exhibiting TG>133 mg/dL- a value within the normal range of TG- usually have an abnormal abundance of sdLDL particles [19]. The NCEP ATP III guidelines for the diagnosis of the MetSyn use as one of the criteria the cut-off point of 150 mg/dL for TG concentration [3]. Therefore, MetSyn subjects, especially those with raised TG levels, might exhibit abnormal abundance of sdLDL particles. Consequently, the inclusion of apoB levels in the calculation of LDL-C could provide a better estimate of LDL-C concentration compared to LDL-F in patients with sdLDL particles. Wagner et al evaluated the accuracy of an apoB-based formula (LDL-W) in type 2 diabetic patients and found that this formula exhibited a bias of -0.5 ± 6.1% against LDL-UC, significantly lower than the bias of LDL-F against LDL-UC (-3.1 ± 6.4%) [20]. The mean difference between LDL-W and LDL-F in the study by Wagner et al was about 1.9 mg/dL, in favor of LDL-W. Our data showed a mean difference between the two equations of about 4.(-30–62) mg/dL in the total cohort, and 7(-30–62) mg/dL and 3(-17–18) mg/dL in MetSyn and nonMetSyn subjects, respectively, in favor of LDL-F. This finding may be explained by the different study populations (diabetic versus MetSyn and nonMetSyn subjects). However, according to the results of the present study, LDL-W did not differ from LDL-F estimation in any study group.

Another study that evaluated LDL-C using an apoB-based formula was that of Planella et al [21]. Planella et al proposed a formula that, according to the results of their study, produces an estimate of LDL-C that better approaches true LDL-C concentrations obtained by ultracentrifugation compared to Friedewald formula, even in patients with IV dyslipidaemia [21]. Our results showed that LDL-P produced an LDL-C estimate that had a mean difference of 14(-16–64) mg/dL, 16(-16–64) mg/dL and 13(-6–27) mg/dL, in the total cohort, in MetSyn and in nonMetSyn groups, respectively, in favor of LDL-F.

LDL-P and LDL-W formulas, which take into account apoB levels, as well as LDL-L, which directly measures the cholesterol of all IDL and LDL subclasses, did not produce different results in MetSyn and nonMetSyn subjects. Moreover, LDL-F was significantly different from LDL-P and LDL-L in the MetSyn group but differed only from LDL-P in the nonMetSyn group. Though our results show that there are not any discrepancies between LDL-P and LDL-L estimations, LDL-L is not suitable for routine use, since it is based on a method that is intended for research use only so far and specifically for the estimation of sdLDL-cholesterol, as well as for the measurement of mean LDL particle size. This finding and the fact that LDL-L was the only LDL-C estimation formula that did not produce different biases in MetSyn and nonMetSyn individuals (while all other formulas produced greater biases in the MetSyn group compared to nonMetSyn group), indicate that none of the currently existing formulas for LDL-C estimation produces accurate results for LDL-C levels in patients with -even mildly- elevated TG levels, as the MetSyn group in the present study. Consequently, it appears that since the reference method (i.e. β-quantification via UC) is expensive, time-consuming and requires special equipment as well as trained personnel for its conduction, that accurate direct methods for the determination of LDL-C levels are required, especially in populations with abnormal lipid and lipoprotein profile.

Finally, we examined the predictive value of the LDL-H/LDL-apoB, proposed by Hattori et al for the evaluation of LDL particle size [22]. According to the results of the present study and using the proposed cut-off point of 1.2 for the above mentioned ratio, the ratio LDL-H/LDL-apoB did not appear to be a sensitive marker of decreased mean LDL particle size, as obtained by Lipoprint LDL System. It should be noted that the normal range of LDL-H/LDL-apoB ratio was derived from a small number of control individuals in the study by Hattori et al.

## Conclusion

Conclusively, the Friedewald formula seems to exhibit some limitations as far as the estimation of LDL-C levels is concerned in patients with the MetSyn. LDL-C levels evaluated by Lipoprint LDL System seem more accurate than other LDL estimation formulas in these patients, since its difference to LDL-F approaches that of the reference method for LDL-cholesterol calculation, i.e. β-quantification. However, Lipoprint LDL System is not applicable to every day clinical practice and its intended use is not for LDL-C estimation. Subsequent research including β-quantification by ultracentrifugation validation is required in order to explore the accuracy of other LDL-C estimation formulas as well as the accuracy of direct methods for LDL-C measurement in patients with the MetSyn who exhibit abnormal abundance of sdLDL particles.

## Materials and methods

### Study population

One hundred and seventy-nine patients attending the Outpatient Lipid Clinic of the University Hospital of Ioannina participated in the present study. Exclusion criteria were: prior atherosclerotic disease (myocardial infarction, unstable angina, ischemic stroke, peripheral arterial disease, percutaneous transluminal coronary angioplasty and coronary artery bypass graft), known diabetes mellitus (fasting glucose ≥ 126 mg/dL), liver disease (serum aminotranferase activity greater than 3-fold, e.g. >120 IU/L, normal range 5–40 IU/L), renal disease (serum creatinine levels greater than 15 mg/L, normal range 6–12 mg/L), hypothyroidism (TSH greater than 5 μIU/ml, normal range 0.5–4.8 μIU/mL). Moreover, patients receiving drugs that could affect lipid metabolism as well as renal or hepatic function were also excluded from the present study. The diagnosis of the MetSyn was based on the NCEP ATP III criteria [3]. The Ethics Committee of the University Hospital of Ioannina gave its approval for the conduction of the study and every participant gave written consent.

### Biochemical parameters

All lipid and lipoprotein determinations were carried out after an overnight fast. Serum levels of TC, HDL-C, and TG were determined enzymatically with an Olympus AU600 automated analyzer (Olympus Diagnostica, Hamburg, Germany). Apolipoproteins (apo) A-I and B were measured with a DADE Behring Nephelometer BN100 (Liederbach, Germany) in total serum. The calculation of LDL-C was conducted according to the following equations:

*Friedewald *(LDL-F) [4]

LDL-F = TC - HDL-C - TG/5 (in mg/dL), excluding patients with TG concentrations ≥ 400 mg/dL.

*Planella *(LDL-P), which focuses on the inclusion of apoB levels in the estimation of LDL-C levels [21]

LDL-P = 0.41*TC - 0.14*TG + 0.66*apoB - 10.43 (in mg/dL).

*Hattori *(LDL-H), an equation very similar to that proposed by Friedewald [22]

LDL-H = 0.94*TC - 0.94*HDL-C -0.19*TG (in mg/dL).

*AMORIS study *(LDL-A), including apoA-I levels in the equation for LDL-C estimation [24]

LDL-A = 18.53 + 0.99*TC - 0.1*TG - 0.61*apoA-I (in mg/dL).

*Wagner *(LDL-W), also including apoB levels in the formula for the estimation of LDL-C concentration [20]

LDL-W = 0.358*TC + 0.776*apoB - 0.149*TG (in mg/dL).

*Lipoprint System *(LDL-L) [25, 26]

The estimation of LDL-C levels was provided by the LDL Lipoprint System, which calculates LDL-C as the sum of the cholesterol carried on IDL and LDL particles. It should be noted that the LDL Lipoprint LDL System's use is not intended for the evaluation of LDL-C levels rather than for the estimation of the cholesterol carried on small, dense LDL (sdLDL) particles, as well as for the estimation of mean LDL particle size.

The mean LDL particle size (in Å) was provided by the LDL Lipoprint System [25]. The ratio LDL-C/LDL-apoB (a proposed index for the estimation of LDL particle size) was calculated according to the formula proposed by Hattori as follows :

LDL-H/LDL-apoB = LDL-H/(apoB-0.09*TC+0.09*HDL-C-0.08*TG) (all lipid constituents measured in mg/dL).

### Statistical analysis

Data are expressed as mean ± SD, except for parameters not following a Gaussian distribution which are expressed AS median (range). Preliminary analysis was performed to ensure no violation of the assumptions of normality and linearity. The Shapiro-Wilk test was used to evaluate whether each variable followed a Gaussian distribution. The relationships between different LDL-C estimations were investigated using Pearson product-moment correlation coefficient. Independent samples t-test (or Mann-Whitney U test when appropriate) was used to assess differences in the various LDL-C estimations as well in the differences and biases of the LDL-calculations in the study groups. One-way analysis of variance (ANOVA) followed by Least Significant Difference (LSD) for paired comparisons were conducted in order to evaluate differences between LDL-C estimations in the total cohort, as well as within each study group. Analysis of covariance (ANCOVA) was performed to evaluate the impact of TG levels on the LDL-C estimations. The sensitivity of LDL-H/LDL-apoB ratio at the specified cut-off point of 1.2 was calculated as: [true positives/(true positives+ false negatives)]*100, where "true positives" means the number of patients with mean LDL particle size ≤ 268Å correctly classified by the LDL-H/LDL-apoB ratio and "false negatives" means the number of patients with mean LDL particle size ≤ 268Å misclassified by the ratio. The specificity of the previously mentioned ratio at the cut-off point of 1.2 was calculated as [true negatives/(true negatives + false positives)]*100, where "true negatives" means the number of patients with mean LDL particle size >268Å correctly classified by the LDL-H/LDL-apoB ratio and "false positives" means the number of patients with mean LDL particle size >268Å misclassified by the ratio. The positive and negative predictive values at the specified cut-off point of 1.2 were calculated as [true positives/(true positives + false positives)]*100 and [true negatives/(true negatives + false negatives)]*100, respectively. A p value of <0.05 was considered to be significant. All analyses were carried out with the SPSS 13.0 softpack.

## Competing interests

The author(s) declare that they have no competing interests.

## Authors' contributions

IG: acquisition of data, LDL Lipoprint System conduction, conception and design of the study, statistical analysis, draft of the manuscript.

VT: statistical analysis, draft of the manuscript.

TDF: acquisition of data, LDL Lipoprint System conduction

VGS: acquisition of data, statistical analysis.

ETB: conception and design of the study, revision of the manuscript

ADT: conception and design of the study, revision of the manuscript

EM: conception and design of the study, supervision and final approval of the submitted manuscript.
